# Oxidative stress mediated cytotoxicity of biologically synthesized silver nanoparticles in human lung epithelial adenocarcinoma cell line

**DOI:** 10.1186/1556-276X-9-459

**Published:** 2014-09-02

**Authors:** Jae Woong Han, Sangiliyandi Gurunathan, Jae-Kyo Jeong, Yun-Jung Choi, Deug-Nam Kwon, Jin-Ki Park, Jin-Hoi Kim

**Affiliations:** 1 Department of Animal Biotechnology, Konkuk University, 1 Hwayang-Dong, Gwangin-gu Seoul 143-701, Korea; 2 GS Institute of Bio and Nanotechnology, Coimbatore, Tamilnadu 641024, India; 3 Animal Biotechnology Division, National Institute of Animal Science, Suwon 441-350, Korea

**Keywords:** Adenocarcinoma cells A549, Reactive oxygen species generation (ROS), Lactate dehydrogenase (LDH), Mitochondrial transmembrane potential (MTP), Silver nanoparticles (AgNP)

## Abstract

The goal of the present study was to investigate the toxicity of biologically prepared small size of silver nanoparticles in human lung epithelial adenocarcinoma cells A549. Herein, we describe a facile method for the synthesis of silver nanoparticles by treating the supernatant from a culture of *Escherichia coli* with silver nitrate*.* The formation of silver nanoparticles was characterized using various analytical techniques. The results from UV-visible (UV-vis) spectroscopy and X-ray diffraction analysis show a characteristic strong resonance centered at 420 nm and a single crystalline nature, respectively. Fourier transform infrared spectroscopy confirmed the possible bio-molecules responsible for the reduction of silver from silver nitrate into nanoparticles. The particle size analyzer and transmission electron microscopy results suggest that silver nanoparticles are spherical in shape with an average diameter of 15 nm. The results derived from *in vitro* studies showed a concentration-dependent decrease in cell viability when A549 cells were exposed to silver nanoparticles. This decrease in cell viability corresponded to increased leakage of lactate dehydrogenase (LDH), increased intracellular reactive oxygen species generation (ROS), and decreased mitochondrial transmembrane potential (MTP). Furthermore, uptake and intracellular localization of silver nanoparticles were observed and were accompanied by accumulation of autophagosomes and autolysosomes in A549 cells. The results indicate that silver nanoparticles play a significant role in apoptosis. Interestingly, biologically synthesized silver nanoparticles showed more potent cytotoxicity at the concentrations tested compared to that shown by chemically synthesized silver nanoparticles. Therefore, our results demonstrated that human lung epithelial A549 cells could provide a valuable model to assess the cytotoxicity of silver nanoparticles.

## Background

Recently, silver nanoparticles (AgNPs) show much interest due to their unique physical, chemical, and biological properties [1]. AgNPs have been widely used in personal care products, food service, building materials, medical appliances, and textiles owing to their unique features of small size and potential antibacterial effect [1-3]. A biological approach to the synthesis of nanoparticles using microorganisms, fungi or plant extracts has offered a reliable alternative to chemical and physical methods to improve and control particle size. When compared to physical and chemical methods, biological method is suitable to control particle size [4,5]. Biological methods have several advantages such as low toxicity, cost-effectiveness, physiological solubility, and stability [4,5].

The use of AgNPs has become more widespread for sensing, catalysis, transport, and other applications in biological and medical sciences. This increased use has led to more direct and indirect exposure in humans [2,6]. AgNPs could induce multiple unpredictable and deleterious effects on human health and the environment due to their increasing use. AgNPs can cause adverse effects in directly exposed primary organs and in secondary organs such as the cardiovascular system or central nervous system (CNS) upon systemic distribution. Nanoparticles can reach the CNS via different routes [7,8]. Elder et al. [9] demonstrated that manganese oxide nanoparticles could reach the brain through the upper respiratory tract via the olfactory bulb in rats. It has been shown that small nanoparticles can translocate through and accumulate in an *in vitro* blood brain barrier model composed of rat brain microvessel vascular endothelial cells [10]. Trickler et al. [11] demonstrated that small nanoparticles could induce inflammation and affect the integrity of a blood-brain barrier model composed of primary rat brain microvessel endothelial cells.

Toxicity of AgNPs depends on their size, concentration, and surface functionalization [12]. A recent report suggested that the size of AgNPs is an important factor for cytotoxicity, inflammation, and genotoxicity [13]. AgNPs have been shown to induce cytotoxicity via apoptosis and necrosis mechanisms in different cell lines [14]. The possible exposure of the human body to the nanomaterials occurs through inhalation, ingestion, injection for therapeutic purposes, and through physical contact at cuts or wounds on the skin [15]. These multiple potential routes of exposure indicate the need for caution given the *in vitro* evidence of the toxicity of nanoparticles. AgNPs have received attention because of their potential toxicity at low concentrations [16]. The toxicity of AgNPs has been investigated in various cell types including BRL3A rat liver cells [17], PC-12 neuroendocrine cells [18], human alveolar epithelial cells [19], and germ line stem cells [20]. AgNPs were more toxic than NPs composed of less toxic materials such as titanium or molybdenum [17].

Several studies reported that AgNP-mediated production of reactive oxygen species (ROS) plays an important role in cytotoxicity [15,20,21]. *In vivo* studies also support that AgNPs induced oxidative stress and increased levels of ROS in the sera of AgNP-treated rats [22]. Oxidative stress-related genes were upregulated in brain tissues of AgNP-treated mice, including the caudate nucleus, frontal cortex, and hippocampus [23]. Many studies have suggested that AgNPs are responsible for biochemical and molecular changes related to genotoxicity in cultured cells such as DNA breakage [15,24]. Stevanovic et al. [25] reported that (l-glutamic acid)-capped silver nanoparticles and ascorbic acid encapsulated within freeze-dried poly(lactide-co-glycolide) nanospheres were potentially osteoinductive, and antioxidative, and had prolonged antimicrobial properties. Several studies also suggest oxidative stress-dependent antimicrobial activity of silver nanoparticles in different types of pathogens [25-27]. Comfort et al. [28] reported that AgNPs induce high quantities of ROS generation and led to attenuated levels of Akt and Erk phosphorylation, which are important for the cell survival in the human epithelial cell line A-431. AgNPs have been more widely used in consumer and industrial products than any other nanomaterial due their unique properties. The most relevant occupational health risk from exposure to AgNPs is inhalational exposure in industrial settings [29]. Therefore, the first goal of this study was to design and develop a simple, dependable, cost-effective, safe, and nontoxic approach for the fabrication of AgNPs of uniform size. This was attempted by treating culture supernatants of *Escherichia coli* treated with silver nitrate. The second goal was the characterization of these biologically prepared AgNPs (bio-AgNPs). Finally, the third goal was to evaluate the potential toxicity of bio-AgNPs and compare them with chemically prepared AgNPs (chem-AgNPs) in A549 human lung epithelial adenocarcinoma cells as an *in vitro* model system.

## Methods

### Chemicals

Penicillin-streptomycin solution, trypsin-EDTA solution, Dulbecco's modified Eagle's medium (DMEM), and 1% antibiotic-antimycotic solution were obtained from Life Technologies GIBCO (Grand Island, NY, USA). Silver nitrate, sodium dodecyl sulfate (SDS), and sodium citrate, hydrazine hydrate solution, fetal bovine serum (FBS), In Vitro Toxicology Assay Kit, TOX7, and 2′,7′-dichlorodihydrofluorescein diacetate (H_2_-DCFDA) were purchased from Sigma-Aldrich (St. Louis, MO, USA).

### Synthesis of bio-AgNPs and chem-AgNPs

Synthesis of bio-AgNPs was carried out according to a previously describe method [4]. Briefly, *E. coli* bacteria were grown in Luria Bertani (LB) broth without NaCl. The flasks were incubated for 21 h in a shaker set at 200 rpm and 37°C. After the incubation period, the culture was centrifuged at 10,000 rpm and the supernatant was used for the synthesis of bio-AgNPs. To produce bio-AgNPs, the culture supernatant treated with 5 mM silver nitrate (AgNO_3_) was incubated for 5 h at 60°C at pH 8.0. The synthesis of bio-AgNPs was monitored by visual inspection of the test tubes for a color change in the culture medium from a clear, light yellow to brown. For comparison with bio-AgNPs, we used a citrate-mediated synthesis of silver nanoparticles to generate chem-AgNPs. The synthesis of chem-AgNPs was performed according to a previously described method [30].

### Characterization of bio-AgNPs

Characterization of bio-AgNPs particles was carried out according to methods described previously [4]. The bio-AgNPs were characterized by UV-visible (UV-vis) spectroscopy. UV-vis spectra were obtained using a Biochrom WPA Biowave II UV/Visible Spectrophotometer (Biochrom, Cambridge, UK). Particle size was measured by Zetasizer Nano ZS90 **(**Malvern Instruments, Limited, Malvern, UK). X-ray diffraction (XRD) analyses were carried out on an X-ray diffractometer (Bruker D8 DISCOVER, Bruker AXS GmBH, Karlsruhe, Germany). The high-resolution XRD patterns were measured at 3 Kw with Cu target using a scintillation counter. (*λ* = 1*.*5406 Å) at 40 kV and 40 mA were recorded in the range of 2*θ* = 5° to 80°. Further characterization of changes in the surface and surface composition was performed by Fourier transform infrared spectroscopy (FT-IR) (PerkinElmer Spectroscopy GX, PerkinElmer, Waltham, MA, USA). Transmission electron microscopy (TEM), using a JEM-1200EX microscope (JEOL Ltd., Akishima-shi, Japan) was performed to determine the size and morphology of bio-AgNPs. TEM images of bio-AgNPs were obtained at an accelerating voltage of 300 kV.

### Cell Culture and exposure to AgNPs

A549 human lung epithelial adenocarcinoma cells were cultured in DMEM medium supplemented with 10% FBS and 100 U/mL penicillin-streptomycin at 5% CO_2_ and 37°C. The medium was replaced three times per week, and the cells were passaged at subconfluency. At 75% confluence, cells were harvested by using 0.25% trypsin and were sub-cultured into 75-cm^2^ flasks, 6-well plates, and 96-well plates based on the type of experiment to be conducted. Cells were allowed to attach the surface for 24 h prior to treatment. A 100 μL aliquot of the cells prepared at a density of 1 × 10^5^ cells/mL was plated in each well of 96-well plates. After culture for 24 h, the culture medium was replaced with medium containing bio-AgNPs prepared at specific concentrations (0 to 50 μg/mL) and chem-AgNPs (0 to 100 μg/mL). After incubation for an additional 24 h, the cells were collected and analyzed for viability, lactate dehydrogenase (LDH) release, and ROS generation according to the methods described earlier [31]. Cells that were not exposed to AgNPs served as controls.

### Cell viability (MTT) assay

The cell viability assay was measured using MTT assay. Briefly, A549 human lung epithelial adenocarcinoma cells were plated onto 96-well flat bottom culture plates with various concentrations of AgNPs. All cultures were incubated for 24 h at 37°C in a humidified incubator. After 24 h of incubation, 10 μL of MTT (5 mg/mL in phosphate-buffered saline (PBS) was added to each well, and the plate was incubated for a further 4 h at 37°C. The resulting formazan (product of MTT reduction) was dissolved in 100 μL of DMSO with gentle shaking at 37°C, and absorbance was measured at 595 nm with an ELISA reader.

### Membrane integrity (LDH release) assay

Cell membrane integrity of A549 human lung epithelial adenocarcinoma cells was evaluated according to the manufacturer's instructions. Briefly, cells were exposed to different concentrations of AgNPs for 24 h and then 100 μL per well of each cell-free supernatant was transferred in triplicate into wells in a 96-well plate, then 100 μL of LDH-assay reaction mixture was added to each well. After 3 h incubation under standard conditions, the optical density was measured at a wavelength of 490 nm using a microplate reader.

### Reactive oxygen species (H_2_-DCFH-DA) assay

A549 human lung epithelial adenocarcinoma cells were cultured in minimum essential medium (Hyclone Laboratories, Logan, UT, USA) containing 10 μM H_2_-DCFDA in a humidified incubator at 37°C for 30 min. Cells were washed in PBS (pH 7.4) and lysed in lysis buffer (25 mM HEPES [pH 7.4], 100 mM NaCl, 1 mM EDTA, 5 mM MgCl_2_, and 0.1 mM DTT supplemented with a protease inhibitor cocktail). Cells were cultured on coverslips in a 4-well plate. Cells were incubated in DMEM containing 10 μM H_2_-DCFDA at 37°C for 30 min. Cells were washed in PBS, mounted with Vectashield fluorescent medium (Burlingame, CA, USA), and viewed with a fluorescence microscope.

### Mitochondrial transmembrane potential (JC-1) assay

The change in mitochondrial transmembrane potential (MTP) was determined using the cationic fluorescent indicator, JC-1 (Molecular Probes Eugene, OR, USA). In intact mitochondria with a normal MTP, JC-1 aggregates have a red fluorescence, which was measured with an excitation wavelength of 488 nm and an emission wavelength of 583 nm using a GeminiEM fluorescence multiplate reader (Molecular Devices, Sunnyvale, CA, USA). By contrast, JC-1 monomers in the cytoplasm have a green fluorescence, which was measured with an excitation wavelength of 488 nm and an emission wavelength of 525 nm. The presence of JC-1 monomers was indicative of a low MTP.

A549 human lung epithelial adenocarcinoma cells were cultured in DMEM containing 10 μM JC-1 in a humidified incubator at 37°C for 15 min. Cells were washed with PBS and then transferred to a transparent 96-well plate. JC-1 monomer-positive cell populations were determined with a FACSCalibur instrument. Cells were cultured on coverslips housed in a 4-well plate, incubated in DMEM containing 10 μM JC-1 at 37°C for 15 min, and then washed with PBS. Cells were mounted with Vectashield fluorescent medium and viewed with a fluorescence microscope.

### Cellular uptake of AgNPs

To study the cellular uptake of AgNPs, cells were treated with AgNPs for 48 h, harvested, and fixed with a mixture of 2% paraformaldehyde and 2.5% glutaraldehyde in 0.2 M PBS for 8 h at pH 7.2. After fixation, the cells were incubated with 1% osmium tetroxide in PBS for 2 h. The fixed cells were dehydrated in ascending concentrations of ethanol (70%, 80%, 90%, 95%, and 100%) and embedded in EMbed 812 resins (EMS, Warrington, PA, USA) via propylene oxide. Ultrathin sections were obtained using an ultramicrotome (Leica, IL, USA) and were double stained with uranyl acetate and lead citrate. The stained sections on the grids were then examined with a H7000 TEM (Hitachi, Chiyoda-ku, Japan) at 80 kV.

## Results and discussion

### Synthesis and characterization of biologically synthesized AgNPs

**Figure 1:**
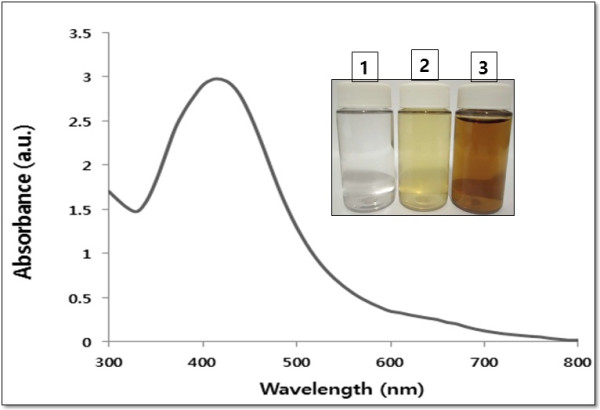
**Synthesis and characterization of bio-AgNPs using culture supernatant from *E. coli.*** The inset shows tubes containing samples of silver nitrate (AgNO_3_) after exposure to 5 h (1), AgNO_3_ with the extracellular culture supernatant of *E. coli* (2), and AgNO_3_ plus supernatant of *E. coli* (3). The color of the solution turned from pale yellow to brown after 5 h of incubation, indicating the formation of silver nanoparticles. The absorption spectrum of AgNPs synthesized by *E. coli* culture supernatant exhibited a strong broad peak at 420 nm and observation of such a band is assigned to surface plasmon resonance of the particles.

The aim of this experiment was to produce smaller size of AgNPs using the culture supernatant of *E. coli* and to understand the effect of toxicity in human lung epithelial A549 cells of the AgNPs. In order to control the particle size of bio-AgNPs, 5 mM AgNO_3_ was added to the culture supernatant and incubated for 5 h at 60°C at pH 8.0 [4,32]. Synthesis was confirmed by visual observation of the culture supernatant. The supernatant showed a color change from pale yellow to brown. No color change was observed during incubation of culture supernatant without AgNO_3_ or in media with AgNO_3_ solution alone (Figure 1 inset). The appearance of a yellowish brown color in AgNO_3_-treated culture supernatant suggested the formation of AgNPs [4,32,33].

Prior to the study of the cytotoxic effect of AgNPs, characterization of bio-AgNPs was performed according to methods previously described [4]. Bio-AgNPs were synthesized using *E. coli* culture supernatant. The synthesized bio-AgNPs were characterized by UV-visible spectroscopy, which has been shown to be a valuable tool for the analysis of nanoparticles [4,34,35]. In the UV-visible spectrum, a strong, broad peak at about 420 nm was observed for bio-AgNPs (Figure 1). The specific and characteristic features of this peak, assigned to a surface plasmon, has been well documented for various metal nanoparticles with sizes ranging from 2 to 100 nm [4,34,35]. In this study, we synthesized bio-AgNPs with an average a diameter of 15 nm.

Next, the cytotoxic effects of bio-AgNPs were evaluated using an *in vitro* model. Earlier studies reported that synthesis of bio-AgNPs by treating the culture supernatant of *E. coli*[4] and *Bacillus licheniformis*[33] with AgNO_3_ produced bio-AgNPs with an average diameter of 50 nm. These bio-AgNPs have been used for both *in vitro* and *in vivo* studies [36-38]. AgNPs with a size of 20 nm or less could enter the cell without significant endocytosis and are distributed within the cytoplasm [39]. Cellular uptake was greater in AgNPs 20 nm or less than with AgNPs above 100 nm in human glioma U251 cells [40]. Park et al. [13] studied the effects of various sizes of AgNPs (20, 80, 113 nm) by testing them in *in vitro* assays such as cytotoxicity, inflammation, genotoxicity, and developmental toxicity. They concluded that for the all toxicity endpoints studied, AgNPs of 20 nm were more toxic than larger nanoparticles.

### XRD analysis of AgNPs

**Figure 2:**
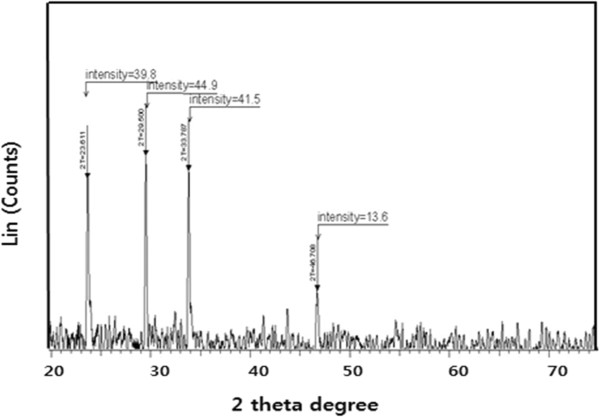
**XRD pattern of AgNPs.** A representative X-ray diffraction (XRD) pattern of silver nanoparticles formed after reaction of culture supernatant of *E. coli* with 5 mM of silver nitrate (AgNO_3_) for 5 h at 50°C. The XRD pattern shows four intense peaks in the whole spectrum of 2*θ* values ranging from 20 to 70. The intense peaks were observed at 2*θ* values of 23.6°, 29.5°, 33.7°, and 46.7°, corresponding to 111, 200, 220, and 311 planes for silver, respectively.

Further characterization was carried out to confirm the crystalline nature of the particles, and a representative XRD pattern of bio-AgNPs is shown in Figure 2. The XRD pattern shows four intense peaks in the whole spectrum of 2*θ* values ranging from 20 to 80. A comparison of our XRD spectrum with the standard confirmed that the silver particles formed in our experiments were nanocrystals, as evidenced by the peaks at 2*θ* values of 23.6°, 29.5°, 33.7°, and 46.7°, corresponding to 111, 200, 220, and 311 lattice planes for silver, respectively. XRD data confirm the crystallization of AgNPs exhibited 2*θ* values corresponding to the previously reported values for silver nanocrystals prepared from the *E. coli* supernatant [4]. Thus, the XRD pattern confirms the crystalline planes of the face-centered cubic (fcc)-structured AgNPs, suggesting the crystalline nature of these AgNPs [4].

### FTIR analysis of AgNPs

**Figure 3:**
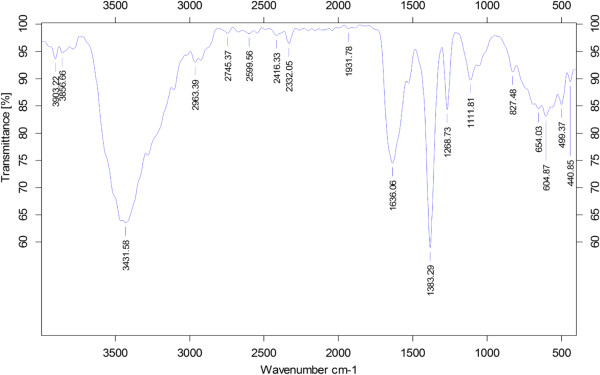
**FT**-**IR spectrum of biologically synthesized silver nanoparticles.**

The FTIR spectrum was recorded for the freeze-dried powder of bio-AgNPs*.* The amide linkages between amino acid residues in proteins give rise to the well-known signatures in the infrared region of the electromagnetic spectrum. The bands between 3,000 and 4,000 cm^−1^ were assigned to the stretching vibrations of primary and secondary amines, respectively, while their corresponding bending vibrations were seen at 1,383 and 1,636 cm^−1^, respectively (Figure 3). The overall spectrum confirms the presence of protein in samples of bio-AgNPs. Earlier studies suggested that proteins can bind to nanoparticles either through their free amine groups or cysteine residues [41]. FTIR provides evidence for the presence of proteins as possible biomolecules responsible for the reduction and capping agent, which helps in increasing the stability of the bio-AgNPs [41].

### Size and morphology analysis of AgNPs by TEM

**Figure 4:**
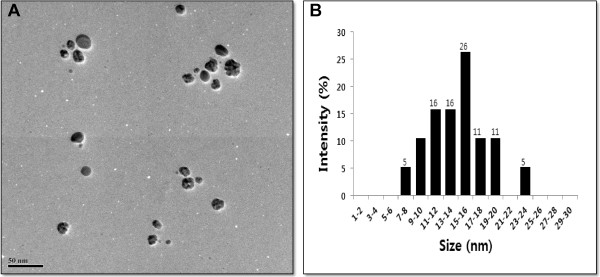
**Size and morphology of AgNPs analysis by TEM. (A)**. Several fields were photographed and used to determine the diameter of silver nanoparticles (AgNPs). **(B)**. Particle size distributions from transmission electron microscopy images. The average range of observed diameter was 15 nm.

TEM is one of the most valuable tools to directly analyze structural information of the nanoparticles. TEM was used to obtain essential information on primary nanoparticle size and morphology [42]. TEM micrographs of the bio-AgNPs revealed distinct, uniformly spherical shapes that were well separated from each other. The average particle size was estimated from measuring more than 200 particles from TEM images and showed particle sizes between 11 and 28 nm with an average size of 20 nm (Figure 4).

Several labs used various microorganisms for synthesis of bio-AgNPs including *Klebsiella pneumonia and E. coli* with an average AgNP size of 52.5 nm and 50 nm, respectively [4,43]. In case of gram-positive bacteria such as *B. licheniformis*[33], *Bacillus thuringiensis*[44], and *Ganoderma japonicum*[45] produced an average size of 50, 15, and 5 nm, respectively. Earlier studies showed that bio-AgNPs synthesized with the supernatant form *E. coli and B. licheniformis* were about 50 nm [4,33]. Interestingly, *E. coli* strain can produce lower sizes of nanoparticles under optimized conditions. Several studies have reported the synthesis of AgNPs using fungi such as spent mushrooms [46], *Pleurotus florida*[47], *Volvariella volvacea*[48], *Ganoderma lucidum*[49], and *Ganoderma neo japonicum*[45]. These AgNPs had average sizes of 20, 15, 45, and 5 nm, respectively. Although various microorganisms produce various sizes, the AgNP size can be adjusted through optimization of various parameters such as concentration of AgNO_3_, temperature, and pH [4].

### Size distribution analysis by dynamic light scattering

**Figure 5:**
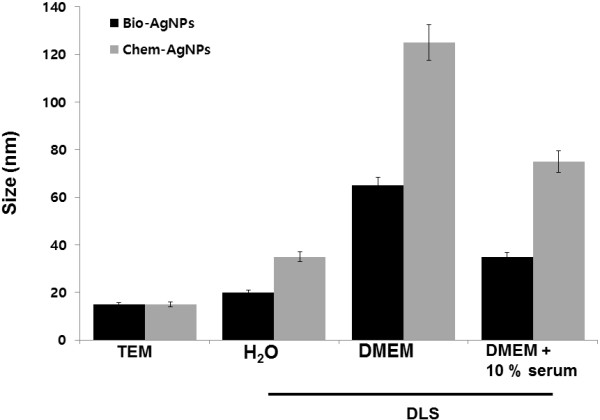
**Size distribution analysis by dynamic light scattering (DLS).** Biologically synthesized silver nanoparticles (bio-AgNPs) and chemically synthesized silver nanoparticles (chem-AgNPs) were dispersed in deionized water and DMEM media with and without serum. The particles were mixed thoroughly via sonication and vortexing, and samples were measured at 25 μg/ml.

TEM images are captured under high vacuum conditions with a dry sample; therefore, additional experiments were carried out to determine particle size in aqueous or physiological solutions using dynamic light scattering (DLS). The characterization of nanoparticles in solution is essential before assessing the *in vitro* toxicity [42]. Particle size, size distribution, particle morphology, particle composition, surface area, surface chemistry, and particle reactivity in solution are important factors in assessing nanoparticle toxicity [42]. Powers et al. [50] proposed DLS as a useful technique to evaluate particle size and size distribution of nanomaterials in solution. In the present study, DLS was used, in conjunction with TEM, to evaluate the size distribution of AgNPs. The bio-AgNPs and chem-AgNPs showed with an average size of 20 and 35 nm, respectively, which is slightly larger than those observed in TEM, which may be due to the influence of Brownian motion. Murdock et al. [42] demonstrated that many metal and metal oxide nanomaterials agglomerate in solution and that, depending upon the solution, particle agglomeration is either stimulated or mitigated. Similarly, we performed size distribution analysis in various solutions such as water, DMEM media, and DMEM with 10% FBS using dynamic light scattering assay. It was found that the average size of bio-AgNPs was 20 ± 5.0, 65 ± 16.0, and 35 ± 8.0 nm in water, DMEM media, and DMEM with 10% serum, respectively. The average size of chem-AgNPs was 35 ± 10.0, 125 ± 20.0, and 75 ± 15.0 nm in water, DMEM media, and DMEM with 10% FBS, respectively (Figure 5). The results suggest that the bio-AgNPs particles dissolved in DMEM media were slightly different from AgNPs dissolved in water. Similarly, DMEM media with 10% FBS showed slight variation in sizes.

DLS results for particle size in solution indicated the chem-AgNPs tended to form agglomerates of greater size than bio-AgNPs when dispersed in either water or cell culture media. The chem-AgNPs particles ranged from 35 nm in water to 125 and 75 nm in DMEM media without and with serum, respectively. Although, both AgNPs were highly agglomerated in DMEM media without serum, the chem-AgNPs agglomeration was significantly greater than bio-AgNPs. This may be due to the type of capping agents used for the synthesis of nanoparticles. Murdock et al. [42] found that Ag-based particles exhibited a similar pattern by agglomerating at nearly the same size when dispersed in either water or media with serum. They also observed that polysaccharide-coated silver nanoparticles with an average size of 80 nm by TEM showed an increase from 250 nm in water to 1,230 nm in RPMI-1640 media with serum.

### Cellular toxicity

These experiments were intended to investigate the cytotoxic effects of bio-AgNPs and chem-AgNPs in lung epithelial adenocarcinoma cells as an *in vitro* model. Viability assays are used to assess the cellular responses of any toxicant that influences metabolic activity [15]. In order to see the effect of AgNPs on cell viability, we used mitochondria function as a cell viability marker in A549 human lung epithelial adenocarcinoma. Incubating bio-AgNPs or chem-AgNPs with medium only and checking the absorption served as the control. These studies showed that the presence of culture media and all the bio-AgNPs/chem-AgNPs did not interfere with the MTT assay.

**Figure 6:**
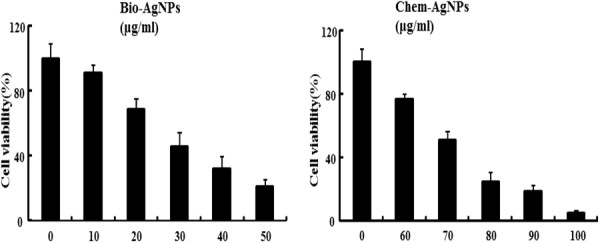
**Effect of AgNPs on cell viability of A549 human lung epithelial adenocarcinoma cells.** Cells were treated with silver nanoparticles (AgNPs) at several concentrations for 24 h and cytotoxicity was determined by the MTT method. The results are expressed as the mean ± SD of three separate experiments each of which contained three replicates. Treated groups showed statistically significant differences from the control group by the Student's *t* test (*p* < 0.05).

Cell viability studies with bio-AgNPs were carried out over the concentration range of 0 to 50 μg/ml. The results suggested that bio-AgNPs at 25 μg/ml decreased the viability of A549 cells to 50% of the control level, so this was determined to be the IC_50_. Exposures to higher concentrations resulted in increased toxicity to the cells (Figure 6). In case of chem-AgNPs, 0 to 50 μg/ml had no toxic effect in A549 cells. We tested additional concentrations between 50 to 100 μg/ml. The results suggested that chem-AgNPs at 70 μg/ml decreased the viability of A549 cells to 50% of the initial level, and this was determined by the IC_50_ (Figure 6).

The MTT cell viability assay demonstrated that both AgNPs produced concentration-dependent cell death. However, chem-AgNPs were less potent in producing cytotoxicity when compared to bio-AgNPs. The less potent cytotoxic effect of chem-AgNPs may be due to higher agglomeration. Uncontrolled agglomeration alters the size and shape of nanoparticles, which greatly influences the cell-particle interactions. Large agglomerations of particles can significantly hinder the effects of individual particle size and shape on toxicity [17]. Zook et al. [51] demonstrated that the large agglomerates of silver nanoparticles caused significantly less hemolytic toxicity than small agglomerates.

Different cytotoxic effects of AgNPs have been reported in various cell types, indicating that AgNPs affected cell survival by disturbing the mitochondrial structure and metabolism [15,52,53]. Our results are in agreement with previous studies about smaller sized AgNPs having been found to be more toxic than larger ones [14,40,44,54]. Mukherjee et al. [55] reported that no inhibition of cell proliferation was observed when A549 cells were incubated with chem-AgNPs (3 and 30 μM).

Gnanadhas et al. [56] demonstrated that the potency of AgNPs was based on the type of capping agent used. Several other studies also reported that capping agents stabilized the AgNPs by decreasing aggregation of the particles and providing protection from temperature and light [57,58]. Enhanced toxicity was observed when AgNPs were coated with different capping agents. Murdock et al. [42] found that the addition of serum to cell culture media had a significant effect on particle toxicity possibly due to changes in agglomeration or surface chemistry. This study was in agreement with earlier reports that suggested that the toxicity of nanoparticles depends on physicochemical properties such as size, shape, surface coating, surface charge, surface chemistry, solubility, and chemical composition [59].

### AgNPs induced LDH leakage

**Figure 7:**
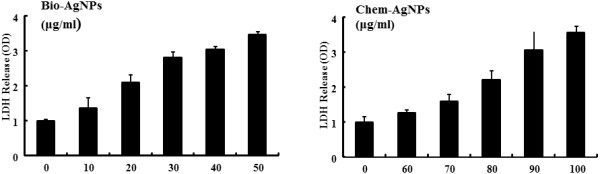
**Effect of AgNPs on LDH release from A549 human lung epithelial adenocarcinoma cells.** Lactate dehydrogenase (LDH) was measured by changes in optical density due to NAD^+^ reduction monitored at 490 nm, as described in the ‘Methods’ section. The results are expressed as the mean ± SD of three separate experiments each of which contained three replicates. Treated groups showed statistically significant differences from the control group by the Student's *t* test (*p* < 0.05).

LDH is an enzyme widely present in cytosol that converts lactate to pyruvate. Release of LDH from cells into the surrounding medium is a typical marker for cell death. When plasma membrane integrity is disrupted, LDH leaks into the media and its extracellular levels increase indicating cytotoxicity by nanoparticles [54] or other substances. We examined whether AgNPs led to LDH leakage into the medium. In order to determine the effect of AgNPs on LDH leakage, the cells were treated with various concentrations of AgNPs and then LDH leakage was measured [31,54]. Cells treated with bio-AgNPs showed significantly higher LDH values in the medium than chem-AgNPs indicate that bio-AgNPs were more potent in producing cytotoxicity in A549 cells (Figure 7). Chem-AgNP-treated cells showed significantly higher LDH release at high concentrations compared to untreated cells (Figure 7).

In this study, the LDH activity in the medium was significantly higher for cells treated with bio-AgNPs, especially at higher concentrations (over 20 μg/mL). Conversely, chem-AgNPs showed toxicity only at higher concentrations (over 60 μg/mL). These findings demonstrated that AgNPs could produce cell death. Miura and Shinohara [60] demonstrated potential cytotoxicity and increased expression levels of stress genes, *ho-1* and *mt-2A*, at higher concentrations of AgNPs in Hela cells. Kim et al. [61] reported size and concentration-dependent cellular toxicity of AgNPs in MC3T3-E1 and PC12 cells. Their studies included assessments of cell viability, reactive oxygen species generation, LDH release, ultrastructural changes in cell morphology, and upregulation of stress-related genes (*ho-1* and *MMP-3*). We found that an IC_50_ concentration of 25.0 μg/mL for bio-AgNPs and 70.0 μg/mL for chem-AgNPs was significant on cell viability. Therefore, these concentrations were used for further studies.

### AgNPs induced generation of ROS

**Figure 8:**
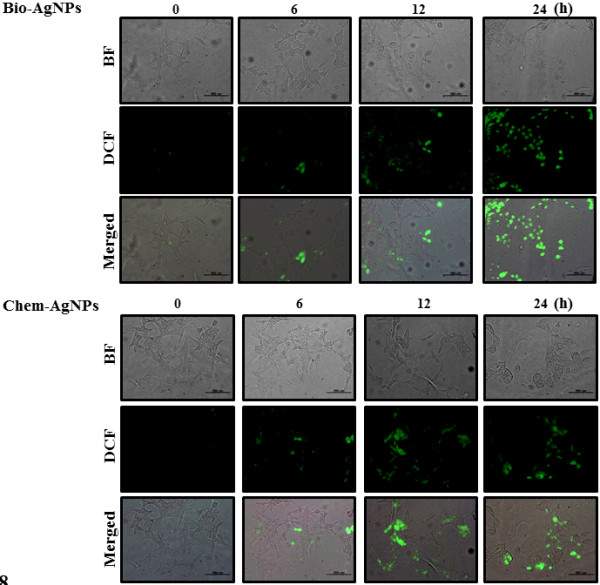
**ROS generation in AgNP-treated A549 human lung epithelial adenocarcinoma cells.** Fluorescence images of A549 cells without silver nanoparticles (AgNPs) (0) and cells treated with biologically synthesized AgNPs (bio-AgNPs) (25 μg/ml) and chemically synthesized AgNPs (chem-AgNPs) (70 μg/ml) and incubated at different time points. Both bio-AgNPs and chem-AgNPs support the formation of hydrogen peroxide inside the A549 cells.

ROS generation is a marker for oxidative stress. Production of ROS causes oxidative damage to cellular components, eventually leading to cell death. Oxidative stress is one of the key mechanisms of AgNPs toxicity and can promote apoptosis in response to a variety of signals and pathophysiological situations [44,54,62,63]. In this assay, we have used DCFH-DA to evaluate ROS production. Figure 8 shows the fluorescence images of untreated A549 cells and cells treated with AgNPs and harvested at different times points. The control sample showed no green fluorescence indicating a lack of H_2_O_2_ formation, whereas bio-AgNP-treated cells showed bright green fluorescence (Figure 8, upper panel). Maximum green fluorescence intensity was observed at 12 and 24 h in A549 cells treated with bio-AgNPs. As shown in Figure 8 (lower panel), untreated A549 cells show much weaker green fluorescence than chem-AgNP-treated cells. More intense green fluorescence was observed with increasing time of incubation. Maximum green fluorescence intensity was observed in the A549 cells treated with bio-AgNPs (25 μg/ml) which exceed the fluorescence produced by chem-AgNPs (70 μg/ml).

A similar trend was seen in the formation of hydrogen peroxide and superoxide anion in the cancer cells treated with bio-AgNPs prepared using *Olax scandens* leaf extract [55]. Several studies have suggested that the antitumor or antiproliferation activity of silver and gold nanoparticles to cancer cells was observed due to formation of ROS inside the cells [45,64-66].

The results of the current study suggested that cells treated with AgNPs showed concentration-dependent ROS production. The generation of ROS can be responsible for cellular damage and eventually lead to cell death. These results are in agreement with previously published results [15,63]. AgNPs treatment generated elevated intracellular ROS levels and abolished antioxidants like reduced glutathione or antioxidant enzymes, such as glutathione peroxidase and superoxide dismutase, leading to the formation of DNA adducts [15,63]. Intracellular ROS were reported to be a crucial indicator of various toxic effects from NPs [53]. Recent studies have reported AgNPs-mediated generation of ROS in different cell types which induced cell death [23,62,67]. Rahman et al. [23] reported that 25 nm sized AgNPs produced a significant increase in ROS production *in vitro* and *in vivo*. The induction of apoptosis by exposure to AgNPs was mediated by oxidative stress in fibroblasts, muscle, and colon cells [62,67]. Recently, Kim et al. [61] showed the production of ROS was detected in both the MC3T3-E1 and PC12 cell lines in a particle size- and concentration-dependent manner.

### Modulation of MTP by AgNPs

**Figure 9:**
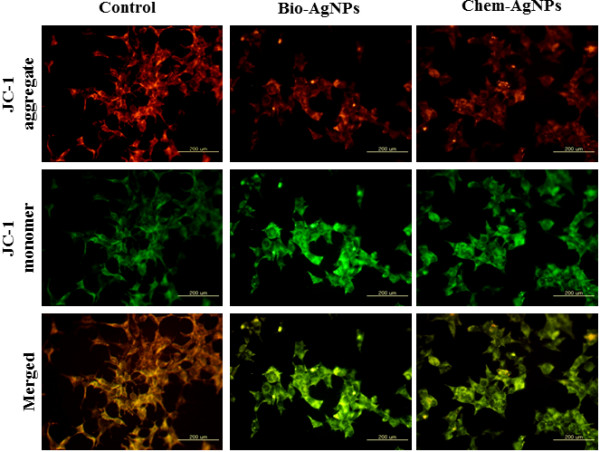
**AgNPs modulates mitochondrial transmembrane potential.** Changes in mitochondrial transmembrane potential (MTP) was determined using the cationic fluorescent indicator, JC-1. Fluorescence images of control A549 cells (without silver nanoparticles (AgNPs)) and cells treated with biologically synthesized AgNPs (bio-AgNPs) (25 μg/ml) and chemically synthesized AgNPs (chem-AgNPs) (70 μg/ml). The changes of mitochondrial membrane potential by AgNPs were obtained using fluorescence microscopy. JC-1 formed red-fluorescent J-aggregates in healthy A549 cells with high MTP, whereas A549 cells exposed to AgNPs had low MTP and, JC-1 existed as a monomer, showing green fluorescence.

Decreased MTP can be an early event in apoptosis. Decreased MTP, as detected by JC-1, was used to investigate whether AgNPs could elicit MTP disruption or not. In general, mitochondria-mediated apoptosis results when mitochondria undergo two major changes. The first change is the permeabilization of the outer mitochondrial membrane, and the second is the loss of the electrochemical gradient [68]. The permeabilization of the outer membrane is tightly regulated by a member of the Bcl-2 family. Membrane depolarization is mediated by the mitochondrial permeability transition pore. Prolonged mitochondrial permeability transition pore opening leads to a compromised outer mitochondrial membrane [68,69]. As shown in Figure 9, the control cells differently exhibited red fluorescence, indicating that a high fraction of mitochondria were in the energized state [70]. However, decreases in mitochondrial energy transduction were observed following treatment of AgNPs for 1 h, illustrated by disappearance of red fluorescence and emergence of green fluorescence. Although both bio-AgNPs and chem-AgNPs could cause MTP collapse, bio-AgNPs were more potent at producing depolarization than chem-AgNPs. These results suggest that AgNPs could induce apoptosis through a mitochondria-mediated apoptosis pathway. A similar observation was made in RAW264.7 cells with the tertbutylhydroperoxide treatment-enhanced mitochondria-mediated apoptosis through failure of MTP [70].

#### Cellular uptake of AgNPs induces accumulation of autophagosomes and autolysosomes

Oxidative stress plays an important role in various pathological conditions including some neurodegenerative diseases and several cardiac diseases which have been related to the process of autophagy [71,72]. Accumulation of ROS, e.g., hydrogen peroxide (H_2_O_2_), is an oxidative stress response, which induces various cell defense mechanisms or programmed cell death [73-75]. Autophagy may protect cells against cell death under oxidative stress, due to higher likelihood that oxidized proteins will be taken up by autophagosomes and subsequently degraded by lysosomes. This process contributes to the efficient removal of oxidized proteins and reduces further oxidative damage [73-76]. Oxidant stress has been implicated in triggering autophagy by certain agents such as hydrogen peroxide (H_2_O_2_) and 2-methoxyestradiol (2-ME) [73-76].

**Figure 10:**
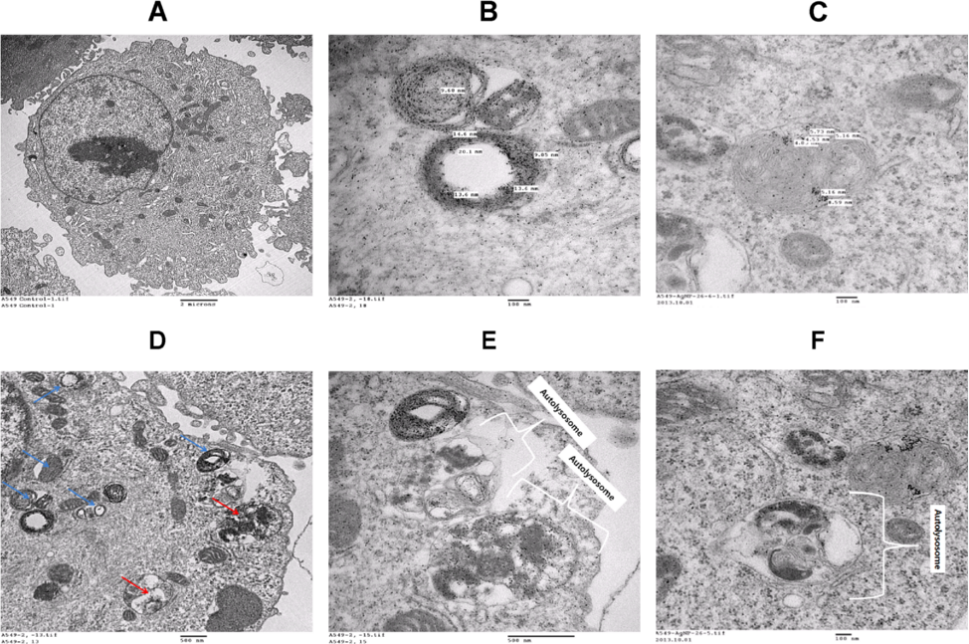
**Intracellular localization of AgNPs and accumulation of autophagosomes and autolysosomes.** A549 cells were treated with silver nanoparticles (AgNPs) for 24 h and then processed for transmission electron microscopy (TEM) sections. TEM images of ultramicrotome slices of A549 cells without AgNPs **(A)**, internalization of biologically synthesized AgNPs (bio-AgNPs) **(B)**, and internalization of chemically synthesized AgNPs (chem-AgNPs) within the cells **(C)**. Bio-AgNPs induces accumulation of autophagosomes (black arrow) and autolysosomes (white arrow) in cells treated with bio-AgNPs for 24 h **(D)**. Autolysosomes and vesicular structures consistent with autophagy were detected in cells treated with bio-AgNPs **(E)** and chem-AgNPs **(F)**.

Cellular uptake studies were carried out to determine the fate of AgNPs in terms of agglomeration, internalization, cell attachment, and accumulation in autophagosomes and autolysosomes. The cells were exposed to the bio-AgNP dose (25 μg/ml) and chem-AgNPs (70 μg/ml) for 24 h, fixed, and prepared for TEM analysis. To avoid misinterpretation due to staining artifacts, the cells were treated with uranyl acetate without lead citrate. The A549 controls appeared normal, with prominent nucleus, nucleolus, and mitochondria (Figure 10A). Bio-AgNP- and chem-AgNPs-treated cells are shown in Figure 10B,C. After 24 h, AgNPs were internalized and cellular morphological changes suggest that autophagy had occurred in the treated cells. AgNPs were localized within membrane-bound cytoplasmic vacuoles and in enlarged lysosomes. Cells exposed to AgNPs exhibit a typical autophagosomes (blue arrow) and autolysosmes (red arrow) with double membranes and enclosed cellular contents (Figure 10D). In addition, when compared to the unexposed control cells, the treated cells showed many multivesicular and membrane-rich autophagosomes in close proximity to each other indicating that AgNPs could induce autophagosome formation at the ultrastructural level. Further, the TEM examination revealed phagophore structures, double-membrane autophagosomes with engulfed damaged organelles (blue arrow), and autolysosomes (red arrow) with an enlarged vacuoles containing large amounts of cellular debris were all present in both bio-AgNPs and chem-AgNPs treated cells (Figure 10E,F). Recently, Deng et al. [77] showed that an increase in cytoplasmic vacuoles in cells exposed to PM2.5 exposure, along with the formation of LC3 puncta and accumulation of LC3-II, the only protein markers reliably associated with completed autophagosomes [77]. Several studies reported that H_2_O_2_ triggered autophagy or apoptosis in U87 cells, HeLa cells, and M14 cells [75,78,79]. Our studies suggest that the accumulation of autophagosomes and enlarged lysomes/autolysosomes may be due to the oxidative stress induced by AgNPs. These preliminary data also suggest that AgNPs may interrupt the autophagic pathway and may have important implications in biomedical applications of nanoparticles. Although these studies have indicated that AgNPs could induce autophagy, further investigation is needed into the detail mechanism of oxidative stress mediated autophagy.

## Conclusions

Silver nanoparticles (AgNPs) have been used in various medical and biomedical applications such as antibacterial, antiproliferative, anticancer, antiangiogenic, and anti-inflammatory. Therefore, this study was designed to evaluate the potential toxicity of bio-AgNPs in human lung epithelial adenocarcinoma cell line (A549). Initially, the biologically synthesized AgNPs were characterized using various analytical procedures using UV-vis spectrometry, XRD, FTIR, TEM, and DLS. The bio-AgNPs were homogenous in shape, and the average size was 15 nm. Cellular toxicity was determined using various cellular assays such as cell viability, leakage of LDH, ROS generation, and mitochondrial membrane potential. ROS generation was significantly increased; there was a strong correlation between the levels of ROS and cell viability. The results suggested that the toxicity of AgNPs was concentration-dependent and bio-AgNPs were significantly more toxic at lower concentrations than chem-AgNPs. Cellular uptake studies revealed that AgNPs entered the cell and eventually induced oxidative stress, and oxidative stress could play a role in the formation of autosomes and autolysosomes in A549 cells. Altogether, our results demonstrated that cell death and autophagy in A549 cells could be mediated through ROS generation induced by AgNPs. These results also suggest that regulation of ROS generation and autophagy might be a potential strategy for treatment of lung cancer.

## Competing interests

The authors declare they have no competing interests.

## Authors’ contributions

JWH and SG performed synthesis of silver nanoparticle (AgNP), design, cell transfection, western blotting, and reverse transcription-polymerase chain reaction (RT-PCR) analysis. JKJ, YJC, and DNK carried out the effect of AgNPs on cell viability and LDH release and performed the statistical analysis. SG, JKP, and JHK wrote the manuscript. J-H Kim supervised the project. All authors discussed the results and commented on the manuscript. All authors read and approved the final manuscript.
